# A multicenter cohort study of primary hypertension in Korea: study design and interim analysis of the Korean registry of target organ damage in hypertension (KorHR)

**DOI:** 10.1186/s40885-017-0072-2

**Published:** 2017-08-02

**Authors:** Suk-Won Choi, Seong Woo Han, Jong Sun Ok, Byung-Su Yoo, Mi-Seung Shin, Sung Ha Park, Kyu-Hyung Ryu

**Affiliations:** 1 Division of Cardiology, Department of Internal Medicine, Dongtan Sacred Heart Hospital, Hallym University, 7 Keunjaebong-Gil, Hwaseong, Gyeonggido 18450 South Korea; 2 Division of Cardiology, Department of Internal Medicine, Yonsei University Wonju Severance Christian Hospital, Wonju, South Korea; 3 Division of Cardiology, Department of Internal Medicine, Gachon University Gil Medical Center, Incheon, South Korea; 4 Division of Cardiology, Department of Internal Medicine, Severance Cardiovascular Hospital, Seoul, South Korea

**Keywords:** Target organ damage, Hypertension

## Abstract

**Background:**

The Korean Registry of Target Organ Damage in Hypertension aims to evaluate the clinical characteristics and prevalence of subclinical target organ damage in Korean hypertensive patients.

**Method:**

This is a prospective, observational, multicenter cohort study in which 23 university hospitals participated. Since May 2013, we have enrolled 1,318 consecutive hypertensive patients without known cardiovascular disease who met the following inclusion criteria: 1) age older than 30 years and 2) the first visit to the participating hospitals was within the last 5 years.

**Results:**

The mean age was 52 ± 12 years; 62.1% were male, and 41.3% were incident hypertensives. Patients with diabetes mellitus accounted for 7.8% of the population and 43.8% had hyperlipidemia or were on statins at baseline. The mean office blood pressures were 152 ± 20/96 ± 14 mmHg for incident hypertensive patients and 129 ± 13/78 ± 10 mmHg for patients on treatment. Patients with electrocardiographic and echocardiographic left ventricular hypertrophy accounted for 18.9 and 25.6%, respectively. The mean brachial-ankle pulse wave velocity (PWV) was 1564 ± 293 m/s and 19.5% had PWV values of more than 1750 cm/s. Patients with microalbuminuria and chronic kidney disease accounted for 21 and 4%, respectively. The first prescribed class of antihypertensive medications was angiotensin converting enzyme inhibitors in 2.9%, angiotensin receptor blockers (ARBs) in 57.5%, diuretics in 7.6%, calcium channel blockers (CCBs) in 61.0%, beta blockers in 17.3%, and fixed dose combination pill in 27.8%.

**Conclusion:**

Our interim analysis shows that subclinical target organ damage in hypertension is considerably present for incident or treated hypertensive patients. CCBs and ARBs were the most commonly prescribed classes of antihypertensive medications and fixed dose combination pills were actively used in Korea.

**Trial registration:**

NCT01861080. Registered 16 May 2013

## Background

Hypertension is an important worldwide public-health challenge because of its high frequency and concomitant risk of cardiovascular and kidney disease [1]. Substantial evidence has established that lowering high blood pressure (BP) is an effective way to prevent cardiovascular and renal diseases [2]. Along with controlling BP, identification and treatment of other cardiovascular risk factors, such as hyperlipidemia, smoking, and diabetes mellitus, is crucial in the management of hypertension.

Another important factors in the risk stratification and management of hypertensive patients are the existence of subclinical target organ damages (SODs) such as left ventricular hypertrophy (LVH), carotid wall thickening, increased pulsed wave velocity (PWV), chronic kidney disease (CKD) with a reduced estimated glomerular filtration rate (eGFR), and microalbuminuria (MAU) [3]. In the European hypertension guidelines, active search for SODs is highly recommended because it allows a better stratification of the cardiovascular risk and helps physicians select the most appropriate antihypertensive medicines [3].

Despite the emphasis of guidelines on active investigation for SOD, large-population based data are limited in terms of the prevalence of SODs in Korean hypertensives. Therefore, we aimed to determine the prevalence of SODs and distinct characteristics in Korean hypertensives by constructing a nationwide hypertension registry. Hereafter, we report the study design and interim analysis of the Korean Registry of Target Organ Damage in Hypertension (KorHR).

## Methods

### Study design

The KorHR is a prospective, observational, multicenter cohort study in which 24 university hospitals in the Republic of Korea participated. From May 2013, patients were consecutively enrolled if they 1) were older than 30 years old, 2) had primary hypertension, and 3) consented to registry enrollment. Study patients were classified into one of two tracks. One is a group of patients who were diagnosed with primary hypertension at enrollment and had never taken an antihypertensive medication (incident group). Patients were also considered as the incident group if they had been diagnosed with primary hypertension before enrollment and had not taken antihypertensive medications for more than 2 weeks from the time of enrollment. The second is a group of patients who had taken antihypertensive treatment before the study enrollment and first visited research institutions within the past 5 years (on-treatment group).

We excluded patients with obvious cardiovascular disease at baseline, such as cerebrovascular disease, coronary artery disease, heart failure, peripheral artery disease, and CKD with an eGFR of less than 30 mL/min/1.73 m^2^.

At the baseline visit, the demographic and anthropometric characteristics of eligible patients were recorded and the medical history, such as diabetes mellitus (DM), hyperlipidemia, regular exercise, and smoking were also reported. DM was defined as fasting plasma glucose ≥ 126 mg/dL or hemoglobin A1c ≥ 6.5% or if patient were taking anti-diabetic medications. Hyperlipidemia was defined as total cholesterol ≥ 240 mg/dL or low density lipoprotein ≥ 160 mg/dL or triglyceride ≥ 200 mg/dL or if patients were taking statins. Regular exercise was defined if patients do exercise (aerobic or anaerobic) for more than 30 min at a time and at least three times a week. The systolic blood pressure (SBP) and diastolic blood pressure (DBP) were measured two or three times in both arms at intervals of 3 min in accordance with the official recommendations for BP measurements [4]. The mean values of each measurement was recorded as the office BPs. Ambulatory BP monitoring was also used to rule out patients with white coat hypertension and evaluate the BP status. Laboratory tests were performed, including simple urinalysis, complete blood count, serum chemistry, serum lipid, serum uric acid, plasma renin activity, and aldosterone. The SODs of hypertension such as LVH, MAU, CKD, and increased PWV, were thoroughly scrutinized.

Finally, we investigated each class of antihypertensive medication that was prescribed first and analyzed a trend of antihypertensive treatment with medicines. We aimed to evaluate the cardiovascular outcomes for up to 5 years, including all cause death, cardiovascular death, ischemic or hemorrhagic stroke, myocardial infarction, coronary revascularization, and heart failure, although we do not report on the cardiovascular outcomes in this interim analysis.

### Definitions of the primary outcome

The primary outcome was the presence of SODs of hypertension, such as LVH, increased PWV, CKD with an eGFR of 30–60 mL/min/1.73 m^2^, and MAU. Electrocardiographic LVH was defined by the modified Sokolow-Lyon index (largest S-wave + largest R-wave > 3.5 mV) and the assessment of echocardiographic LVH was based on the criteria proposed by the European hypertension guidelines [left ventricular mass index: men >115 g/m^2^ and women >95 g/m^2^ (BSA)] [3]. Left ventricular mass was calculated according to linear method using the following equation [5].

Left ventricular mass = 0.8 * 1.04 * [(thickness of interventricular septum at diastole + left ventricular internal diameter at diastole + posterior wall thickness at diastole)^3^ – (left ventricular internal dimension at diastole)^3^] + 0.6 g.

The calculation of relative wall thickness (RWT) by the formula [(2*posterior wall thickness at diastole)/left ventricular internal dimension at diastole] permits the categorization of LVH as either concentric (RWT > 0.42) or eccentric (RWT ≤ 0.42) [5]. MAU was defined as a urine albumin/creatinine ratio (ACR) in spot urine of ≥30 mg/g but <300 mg/g [6]. Individuals with an eGFR ≤ 60 mL/min per 1.73 m^2^ are defined as having CKD according to the K/DOQI clinical practice guidelines for CKD [7]. We calculated the eGFR using the Modification of Diet in Renal Disease Study equation [8]. The brachial-ankle pulse wave velocity (baPWV) was measured with the individuals in a supine position after at least 5 min of rest. Pressure waveforms of the brachial and tibial arteries were simultaneously recorded by placing occlusion cuffs connected to a plethysmographic sensor around both the brachia and ankles. The time delays (*T*) of the two waveforms between one foot and the other were measured. The lengths of the paths from the suprasternal notch to the brachium (Lb) and from the suprasternal notch to the ankle (La) were automatically calculated according to the height of each individual. The baPWV was calculated using the following equation:

$$ \mathrm{baPWV}=\frac{\left(\mathrm{La}-\mathrm{Lb}\right)}{T\left(\frac{m}{s}\right)} $$

A threshold of >1,750 cm/s for the baPWV was determined for the presence of SOD, which was the cut-off value of baPWV for predicting cardiovascular disease in previous studies [9, 10].

### Statistical analysis

Continuous variables were expressed as the mean ± standard deviation (SD) and were compared using the *t* test or Wilcoxon’s rank-sum test. Categorical data were expressed as frequencies and percentages, and they were compared using the *χ* ^2^ test or Fisher’s exact test. Continuous variables were compared using the student’s *t* test or by the nonparametric Mann-Whitney *U* test. For all tests, a probability value of *p* <0.05 was considered significant. All statistical analyses were performed using statistical software SPSS (Chicago, Illinois) version 19.0.

## Results

**Fig. 1:**
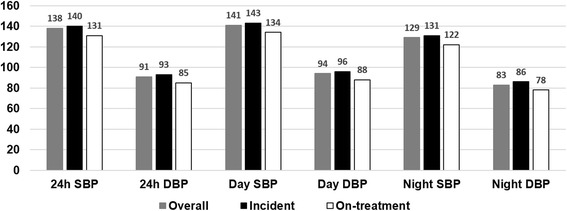
Values of Twenty-four hour ambulatory blood pressure monitoring. 24 h, 24 h average; SBP, systolic blood pressure; DBP, diastolic blood pressure; Day, awake time average; Night, sleep time average

As of April 2016, a total of 1,318 patients with primary hypertension had been enrolled in the KorHR registry. Of 1,318 eligible patients, 611 patients (46%) were included in the incident group and the remaining 707 patients (54%) were in the on-treatment group. Demographic and anthropometric characteristics of the patients are described in Table 1. In short, the mean age of all patients was 51 years and 60% of the patients were male. Patients with obesity (BMI ≥25 kg/m^2^) accounted for 57.5%. Hyperlipidemia (43.8%) was the most common comorbidity. The mean overall office BP was 140 ± 20/86 ± 15 mmHg. Among patients in the on-treatments group, 534 (75.5%) subjects had a BP that was lower than 140/90 mmHg. Table 2 shows the baseline laboratory data. Approximately 60% of study patients had impaired fasting glucose (fasting glucose more than 100 mg/dL but less than 126 mg/dL). The average eGFR was 89 ± 20 ml/min/1.73 m^2^ (BSA) and mean LDL was 114 ± 33 mg/dl. The mean baPWV was 1558 ± 285 cm/s in all patients. In Fig. 1, 24-hour ambulatory BP monitoring is shown. Of 1,318 patients, 711 performed 24-hour ambulatory BP monitoring. The mean 24-hour SBP and DBP were 138 and 91 mmHg, respectively.

**Table 1:** Baseline characteristics

	Overall (*N* = 1,318)	Incident (*N* = 611)	On treatment (*N* = 707)
Age, years ± SD	52 ± 12	47 ± 11	56 ± 11
Males (%)	818 (62.1)	415 (67.9)	403 (57.0)
BMI^a^ (kg/m^2^) ± SD^b^	25.9 ± 3.5	26.2 ± 3.6	25.8 ± 3.4
Obesity^c^ (%)	56.8	61.2	52.8
Waist (cm) ± SD	88 ± 9	89.7 ± 8.8	87 ± 9
Office SBP (mmHg) ± SD	140 ± 20	152 ± 20	129 ± 13
Office DBP (mmHg) ± SD	86 ± 15	96 ± 14	78 ± 10
Office HR (beats per minute) ± SD	75 ± 18	77 ± 12	72 ± 11
Hypertension awareness (months)	48 ± 65	22 ± 40	71 ± 74
Diabetes mellitus (%)	7.8	3.9	11.0
Hyperlipidemia (%)	43.8	35.1	51.0
Regular exercise (%)	32.3	28.8	39.8
Smoking (%)			
Never	54.8	51.5	57.4
Past	19.4	15.1	22.8
Current	20.2	25.6	15.8

^a^ *BMI* body mass index; ^b^ *SD* standard deviation; ^c^Obesity defined as a BMI ≥25 kg/m^2^

**Table 2:** Laboratory Data

	Overall (*N* = 1,318)	Incident (*N* = 611)	On treatment (*N* = 707)
Hemoglobin (g/dL)	14.6 ± 3.5	14.9 ± 1.6	14.4 ± 4.6
Hematocrit (%)	43.0 ± 4.5	44.0 ± 4.8	42.1 ± 3.9
Glucose (mg/dL)	108 ± 25	106 ± 24	109 ± 26
Impaired fasting glucose (%)	59.8	56.0	63.0
Total cholesterol (mg/dL)	188 ± 36	200 ± 36	177 ± 32
Triglyceride (mg/dL)	151 ± 109	166 ± 128	139 ± 87
HDL (mg/dL)	52 ± 14	51 ± 15	52 ± 13
LDL (mg/dL)	114 ± 33	127 ± 32	104 ± 31
Uric acid (mg/dL)	5.5 ± 2.4	5.8 ± 3.2	5.3 ± 1.4
Estimated GFR (mL/min/1.73 m^2^)	89 ± 20	93 ± 26	87 ± 21
Urine albumin creatinine ratio (mg/g), median (1/4 quartile, 3/4 quartile)	10.3 (4.9, 24.8)	10.5 (5.0, 23.7)	10.1 (4.8, 25.5)
Renin (ng/ml/h)	3.35 ± 6.75	3.12 ± 6.76	4.16 ± 6.71
Aldosterone (pg/mL)	9.8 ± 7.5	9.6 ± 6.5	10.42 ± 10.05
PWV^a^	1558 ± 285	1569 ± 289	1543 ± 278
Central SBP	133 ± 20	136 ± 19	130 ± 21
Central DBP	88 ± 13	92 ± 12	83 ± 12
Augmentation index	38 ± 27	33 ± 24	47 ± 28

^a^ *PWV* pulsed wave velocity

**Fig. 2:**
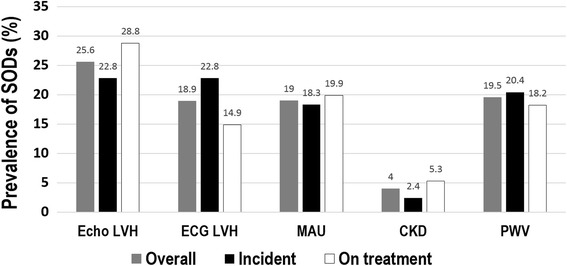
Prevalence of subclinical target organ damage. SOD, subclinical organ damage; Echo LVH, echocardiographic left ventricular hypertrophy; ECG LVH, electrocardiographic left ventricular hypertrophy; MAU, microalbuminuria; CKD, chronic kidney disease; PWV, pulse wave velocity

The prevalence of SODs, which is the primary endpoint of this study, is shown in Fig. 2. Echocardiography was performed in 524 patients and 1,088 patients underwent electrocardiography. The prevalence of echocardiographic and electrocardiographic LVH were 25.6 and 18.9% in all patients, respectively. When comparing the incident group with the on-treatment group, although the overall echocardiographic LVH seemed to be more prevalent in the on-treatment group (28.8 vs. 22.8%, *p* = 0.132), it was not statistically significant (Table 3). In terms of hypertensive ventricular remodeling, eccentric LVH was significantly more frequent in the on-treatment group than the incident group (18.9 vs. 10.0%, *p* = 0.004). However, electrocardiographic LVH was significantly more frequent in the incident group than the on-treatment group (22.8 vs. 14.9%, *p* = 0.015). The test of MAU using ACR was performed in 940 patients. Among them, 179 patients (19.0%) had MAU and 26 patients (2.1%) had overt proteinuria. The value of the eGFR could be obtained in 1,161 patients and the prevalence of CKD, which was defined as an eGFR 30–60 ml/min/1.73 m^2^ (BSA), was 4%. When we set the cut-off value of baPWV to 1,750 m/s, the prevalence of an increased PWV was 19.5%.

**Table 3:** Echocardiographic left ventricular hypertrophy

	Overall (*N* = 524)	Incident (*N* = 281)	On treatment (*N* = 243)	*p* value
LVH	25.6	22.8	28.8	0.132
Concentric LVH (%)	11.5	12.8	9.9	0.336
Eccentric LVH (%)	14.1	10.0	18.9	0.004
Concentric remodeling (%)	12.2	12.1	12.3	0.932
Normal (%)	62.2	65.1	58.8	0.149

The class of antihypertensive medicines prescribed at baseline was investigated. The most frequently prescribed class was calcium channel blockers (CCBs) (61%), which was followed by angiotensin receptor blockers (ARBs) (57.5%), beta blockers (17.6%), diuretics (7.6%), and angiotensin converting enzyme (ACE) inhibitors (2.9%). Among them, 27.8% were prescribed with a fixed dose combination pill; most of these combination pills were an ARB plus CCB (83.6%).

## Discussion

This study shows the integrated prevalence of SODs for primary hypertension in hypertensive patients in Korea. SODs in primary hypertension were quite prevalent: 18.9% for ECG LVH, 25.6% for echo LVH, 19% for MAU, and 19.5% for increased PWV.

With respect to the LVH, eccentric LVH (14.1%) was slightly more frequent than concentric LVH (11.5%) in all subjects. Notably, although concentric LVH seemed to be more common in the incident group than in the on-treatment group, albeit not statistically significant, eccentric LVH was significantly more frequent in the on-treatment group than in the incident group. This finding is consistent with previous studies, including a recent systemic review on echocardiographic LVH [11, 12]. Mancia et al. reported that eccentric LVH was more frequent than concentric LVH and the change in the left ventricular geometry due to high BP was retained regardless of whether the BP was normal or elevated and whether treatment did or did not achieve BP control [11]. As mentioned above, approximately 75% of patients in the on-treatment group in this study achieved target BP. As they proposed, the persistence of cardiac structure abnormalities such as LVH may not completely regress even after achieving target BPs. Additionally, the structural geometry may change with treatment, i.e., from concentric LVH to eccentric LVH with alteration of the RAS system. Concentric LVH was associated with a worse prognosis than the eccentric LVH [13]. However, eccentric LVH was also a significant predictor of the development of low LVEF. [14] Therefore, this result showed that even incident hypertensive patients are exposed to a risk of cardiovascular diseases in addition to patients on hypertensive treatment regardless of whether they achieved target BP. In contrast to echocardiographic LVH, electrocardiographic LVH was more prevalent in the incident group than in the on-treatment group. This finding can be explained that electrocardiographic change due to LVH and BP control might occur more easily than an echocardiographic left ventricular mass change.

MAU is known to be the integrated marker of SODs in primary hypertension [15]. MAU is associated with metabolic derangements and an increased cardiovascular risk in hypertension patients [16, 17]. The previously reported prevalence of MAU varies from 4.7 to 40% according to study populations [18–23]. Among them, the I-DEMAND (Italy Developing Education and awareness on Microalbuminuria in hypertensive Disease) study, which enrolled patients with similar characteristics as this study, showed that the overall prevalence of MAU was 27% in 3,534 hypertensive patients [19]. Two previous Korean studies showed that the prevalence of MAU were 5.4 and 10.1%, which included patients who visited primary clinics or participated in the health examination program [22, 23]. Several factors are known for affecting the prevalence of MAU. MAU is more frequent in patients with high stages of hypertension [24]. In addition to the severity of hypertension, age, sex, obesity, and concomitant risk factors could influence the presence of MAU, although other studies report conflicting results [20, 25–27]. The difference between the present study and other Korean studies is mainly due to the characteristics of patients who visited different clinical settings, such as primary clinics and referral hypertension specialized clinics. The result suggests that MAU is significantly prevalent in Korean hypertensive patients and active investigation for MAU is encouraged for all hypertensive patients, as recommended by the guidelines. Although the prevalence of asymptomatic CKD was rare in incident hypertensives (2.4%), it rose to a considerable level (5.3%) in patients who have been treated with antihypertensive medicines for up to 5 years.

The carotid-femoral PWV (cfPWV) has been acknowledged as the standard of arterial stiffness [28]. However, the baPWV, which is much easier to measure, is more available in South Korea. Previous reports have shown that baPWV was correlated well with cfPWV and predicted worse outcomes in hypertensive patients [29, 30]. These studies suggest that the baPWV cut-off value was approximately 1750 cm/s, which was associated with increased cardiovascular events and mortality [9, 10, 31, 32]. In this study, an overall prevalence of baPWV of more than 1750 cm/s was 11.9%. The prevalence of increased baPWV was significantly higher in the incident group than in the on-treatment group (16.0% vs. 8.3%, *p* < 0.001), which could probably be affected by the high BP status of the incident group.

This study has limitations. First, we enrolled patients who visited hypertension specialized clinics in university hospitals. Because these patients could not be extrapolated to general population with hypertension, the prevalence of SODs could be exaggerated. Second, the performance rate of ECG, MAU, PWV, and 2D echocardiography was 82, 71, 61 and, 40%. Therefore, the findings of 2D echocardiography could be skewed. However, the prevalence of each SOD was quite comparable to previous studies.

Finally, our analysis of antihypertensive medicines demonstrated that CCBs and ARBs are the most commonly prescribed antihypertensive medicines. Notably, ACE inhibitors were rarely prescribed, which is probably due to the high incidence of ACE inhibitor-induced cough in Asians [33]. Furthermore, fixed dose combination pills, which are expected to improve drug compliance, are actively used in South Korea. [34] In South Korea, there is no commercially available fixed dose combination pill that incorporates both an ACE inhibitor and CCB, which could explain the high rate of ARB uses.

## Conclusions

Interim analysis of the KorHR study showed that SODs of hypertension were prevalent in both incident and on-treatment hypertensive patients. In terms of medicines, CCBs and ARBs were the most commonly prescribed classes of antihypertensive medications and fixed dose combination pills are actively used in South Korea.
